# A high performance flexible two dimensional vertically aligned ZnO nanodisc based piezoelectric nanogenerator *via* surface passivation†

**DOI:** 10.1039/c9na00789j

**Published:** 2020-03-20

**Authors:** Ketki Verma, Dhiraj Kumar Bharti, Simadri Badatya, Avanish Kumar Srivastava, Manoj Kumar Gupta

**Affiliations:** Advanced Construction Materials Division, CSIR-Advanced Materials and Processes Research Institute Bhopal Madhya Pradesh 462026 India mkgupta@ampri.res.in manojampri@gmail.com; Academy of Scientific and Innovative Research (AcSIR) Bhopal Madhya Pradesh 462026 India

## Abstract

Herein, we present the growth of pristine vertically aligned flexible two dimensional (2D) pure ZnO nanodiscs *via* a simple seed assisted solution route and their use in the fabrication of a piezoelectric nanogenerator. The preferred growth direction and morphology of wurtzite ZnO nanodiscs were investigated using X-ray diffraction and field emission scanning electron microscopy (FESEM) studies. A flexible piezoelectric nanogenerator was fabricated using the vertically aligned ZnO nanodiscs as the active piezoelectric material and a carbon nanotube–polydimethylsiloxane (CNT : PDMS) film as the top electrode. This unique 2D-type ZnO nanodisc-based nanogenerator generated a direct current (DC) type output voltage and current density of about 2.5 V and 30 nA cm^−2^ under compressive vertical strain, respectively. Significant enhancement of the piezoelectric output voltage from the flexible nanogenerator based on the vertically aligned two dimensional (2D) zinc oxide (ZnO) nanodiscs was achieved *via* thermal annealing. An output voltage and current density of 17 V and 150 nA cm^−2^ were detected from the thermally annealed 2D ZnO nanodisc based nanogenerator which is approximately 8 times higher (voltage) than that from the pristine nanogenerator. It is proposed that the output performance of the vertically aligned ZnO nanodisc based nanogenerators increases due to surface passivation and reduction of oxygen vacancies.

## Abstract 2

Herein, we present the growth of pristine vertically aligned flexible two dimensional (2D) pure ZnO nanodiscs *via* a simple seed assisted solution route and their use in the fabrication of a piezoelectric nanogenerator.

## Graphical abstract

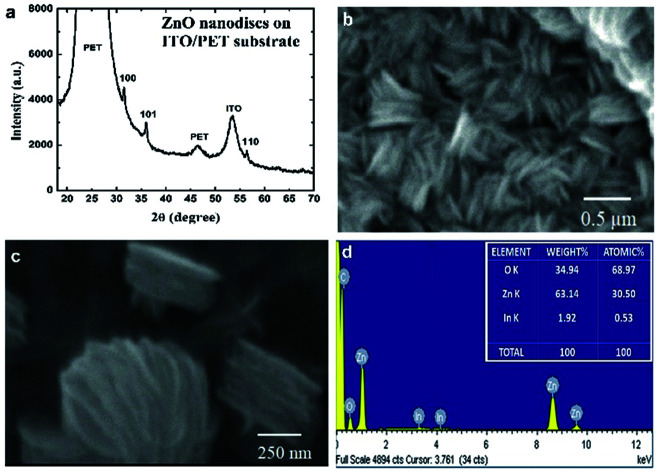

## Introduction

Piezoelectric nanostructures have attracted tremendous interest due to their advanced technological applications including harvesting mechanical energy from the living environment and designing self-powered nanoscale and small scale electronics devices.^1^ In particular, piezoelectric nanogenerators are of intense interest since they can be alternative sources of energy for developing sustainable and green energy for self-powered nanosystems and self-powered nanosensors including wireless nanosensors, gas sensors, mercury detection real-time sensors, implantable biological devices, environmental monitoring and personal electronics and self-powered water splitting systems.^2,3^ Development of such nano power systems dramatically reduces the size of the integrated assembly of nanosystems for optoelectronics, biosensors and resonators.^4^ Piezoelectric materials with semiconductor properties are the key materials for fabricating piezoelectric nanogenerators for harvesting mechanical energy and also for tuning the performance of solar cell/photovoltaic devices through piezophotonic applications.^5^ The structure of ZnO can be described as a number of alternating planes composed of tetrahedrally coordinated O^2−^ and Zn^2+^ ions, and creation of a dipole moment along the c-axis leads to piezoelectricity in ZnO. One-dimensional ZnO nanorods/nanowires have been extensively studied for their application in field emission devices, light emitters, solar cells, electroluminescent devices, laser diodes, photocatalysis photoluminescence, optical lasers, *etc.* Moreover, flexible piezoelectric nanogenerators based on n-type and p-type ZnO nanorods, thin films, nanopowders and nanowires have been reported by various researchers.^6,7^ To synthesize these nanostructures, various methods such as chemical vapor deposition (CVD), hydrothermal and sol gel methods, r-f sputtering, and physical vapor deposition have been employed.^8–11^ Besides one-dimensional (1-D) ZnO nanoparticles, recently, two-dimensional (2-D) ZnO nanostructures have emerged as important nanostructures in the fabrication process of nanoscale devices and sensors owing to their unique new and dramatic physical, chemical and electronic properties including a high surface-to-volume ratio, fast response and good mechanical durability for application in energy conversion and storage devices.^12^ However, 2D ZnO nanostructures are rarely utilised to fabricate nanogenerators because of their difficult and complicated growth process. Recently, Zhang *et al.* reported the fabrication of nanogenerators using ZnO nanosheet networks and an output voltage of 0.924 V was reported.^13^ Similar efforts have also been made using different ZnO nanostructures. Qiu. Y *et al.* reported a flexible ZnO nanorod based nanogenerator with an output voltage of 10 mV and G. Zhu *et al.* synthesized a lateral ZnO nanowire array to fabricate a piezoelectric nanogenerator and they achieved an output voltage of around 2.03 V.^14,15^ It has been reported that the diverse growth pattern of ZnO nanoparticles can be altered by varying the synthesis process parameters and growth conditions such as temperature, pH and precursor concentration. In this regard, B. Kumar, *et al.* synthesized 2D ZnO nanosheets and demonstrated a DC type piezoelectric nanogenerator using nanowire–nanowall hybrid ZnO structures; however the piezoelectric output voltage/current was quite low (∼20 mV).^16^ It has been reported that oxygen vacancies, OH^−^ ions, and high electron carrier density have been invoked as potential factors contributing to n-type conductivity and reducing the piezoelectric power performance of nanogenerators.^17^ Recently, 2D materials such as MoS_2_ nanosheet and bilayer WSe_2_ based piezoelectric nanogenerators have also been reported.^18–21^ To improve the power efficiency of ZnO based nanogenerators, hybridization with a p-type polymer such as poly(3-hexylthiophene-2,5-diyl) (P3HT) and poly(3,4-ethylenedioxythiophene) polystyrene sulfonate (PEDOT:PSS) was employed.^22,23^ Moreover, doping transition and alkali elements in ZnO also increased the piezoelectric charge coefficient and nanogenerator performance. However, controlling the morphology without doping and maintaining the piezoelectric properties are challenging tasks. In this work, we have synthesized 2D vertically aligned piezoelectric ZnO nanodiscs grown on a flexible and transparent indium tin oxide (ITO) coated polyethylene terephthalate (PET) substrate using a seed assisted low cost chemical method. The crystal structures and morphology of vertically aligned ZnO nanodiscs were measured using X-ray diffraction and field emission scanning electron microscopy (FE-SEM) techniques. The piezoelectric nanogenerator was fabricated using 2D ZnO nanodiscs and a carbon nanotube–polydimethylsiloxane (PDMS) composite layer as the top electrode. The output voltage from the vertically aligned flexible ZnO nanodisc nanogenerator was measured under vertical compressive stress. The output performance was dramatically improved by thermal annealing of the 2-D ZnO nanodisc based nanogenerator and an output voltage and current density of 17 V and 150 nA cm^−2^ were obtained, respectively.

## Results and discussion

**Fig. 1:**
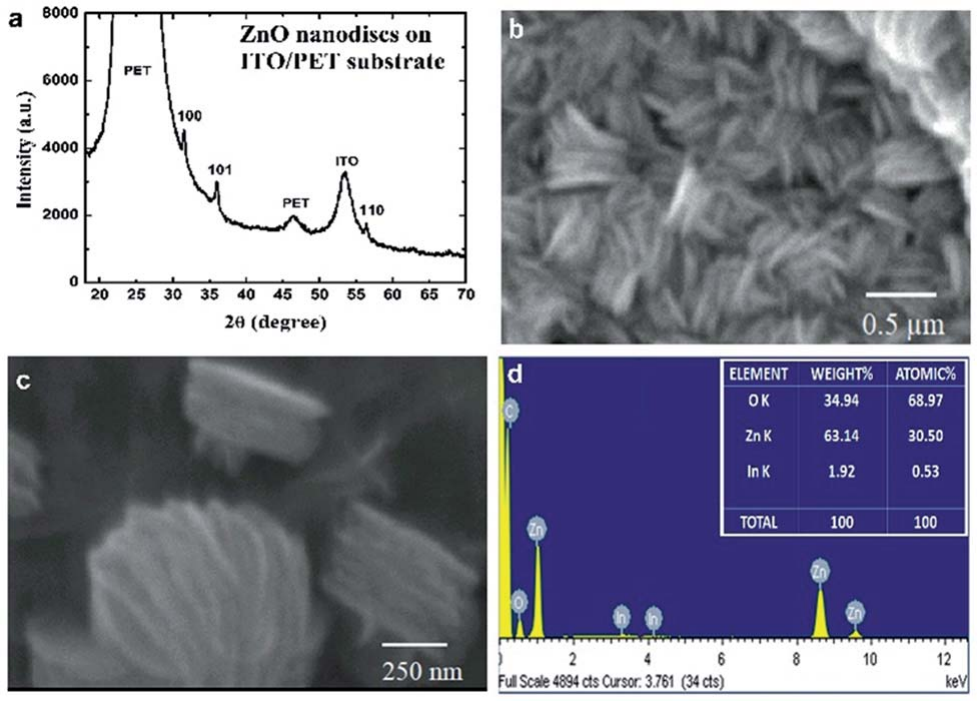
(a) X-ray diffraction pattern of vertically aligned 2D ZnO nanodiscs on the ITO/PET substrate, (b and c) FE-SEM images and (d) EDS spectra of the synthesized ZnO nanodiscs.

XRD analysis was performed to study the structural properties of the as-grown flexible ZnO nanodiscs on the ITO coated PET substrate. Fig. 1a shows the XRD pattern of the ZnO nanodisc sample. The hexagonal wurtzite crystal structure of ZnO grown on the flexible ITO/PET substrate was confirmed by XRD analysis. It was noted that the prominent peaks observed in Fig. 1a are (100), (101) and (110) and the intensity of the diffraction peak corresponding to the (002) plane was very weak, confirming the formation of ZnO along the *a*-axis. To further confirmed the formation of ZnO, we have also recorded the XRD pattern of ZnO nanodisc powder collected from the ITO/PET substrate and this XRD pattern is shown in Fig. S1.† It is clear that all the characteristic peaks along with the (002) plane are present in the powder XRD pattern. These analysis results further confirmed the formation of hexagonal ZnO nanodiscs (JCPDS Card: 36-1451).^24^ The crystallite size of ZnO nanodiscs was calculated using the Debye–Scherrer equation, *D* = *Kλ*/(*β* cos *θ*), where *D* is the mean size of crystallites (nm), *K* is the crystallite shape factor and has a value of about 0.9, *λ* is the X-ray wavelength (1.54060 Å), *β* is the full width at half the maximum (FWHM) in radians of the X-ray diffraction peak and *θ* is Bragg's angle. The average crystallite size of vertically aligned ZnO nanodiscs was estimated and found to be 36 nm.

Morphological analysis of ZnO nanodiscs was performed using the FE-SEM technique. The recorded images are shown in Fig. 1b and c. The vertically aligned 2D nanodisc like morphology of ZnO is depicted in the FE-SEM images. The average thickness and diameter of ZnO nanodiscs was measured and found to be 30 nm and in the 1.0–1.5 μm range, respectively (Fig. 1b and c). Elemental analysis was performed by EDS coupled with SEM. The EDS pattern is shown in Fig. 1d. It is clearly seen that energy peaks corresponding to zinc and oxygen are present and a peak corresponding to the indium element was also detected.

**Fig. 2:**
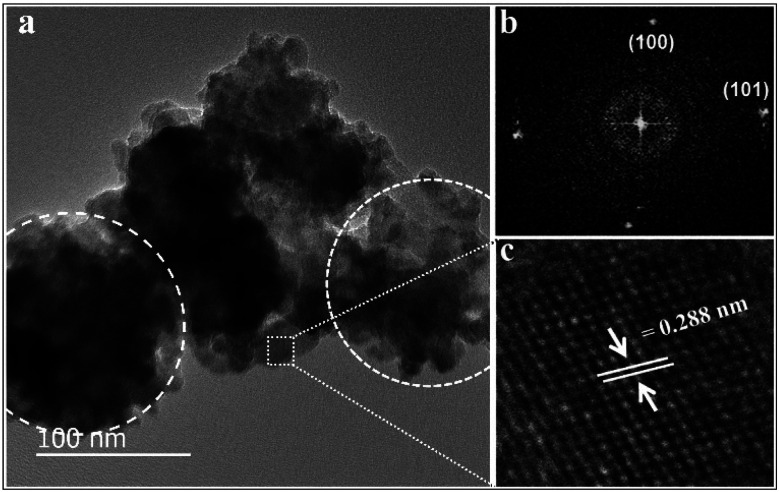
(a) TEM micrograph of stacked ZnO nanodiscs, (b) FFT pattern of ZnO nanodiscs and (c) HR-TEM micrograph showing the lattice fringe image for the (100) plane.

The crystalline quality and morphology of ZnO nanodiscs were investigated using HR-TEM analysis. Fig. 2 shows the TEM images of ZnO nanodiscs, which indicate the formation of the nanodisc like morphology. The fast Fourier transform pattern (FFT) of the high-resolution TEM image of nanodiscs is shown in Fig. 2b. Fig. 2c reveals clear lattice fringes with a *d*-spacing of 0.288 nm corresponding to the (100) plane of the hexagonal wurtzite ZnO crystal.

**Fig. 3:**
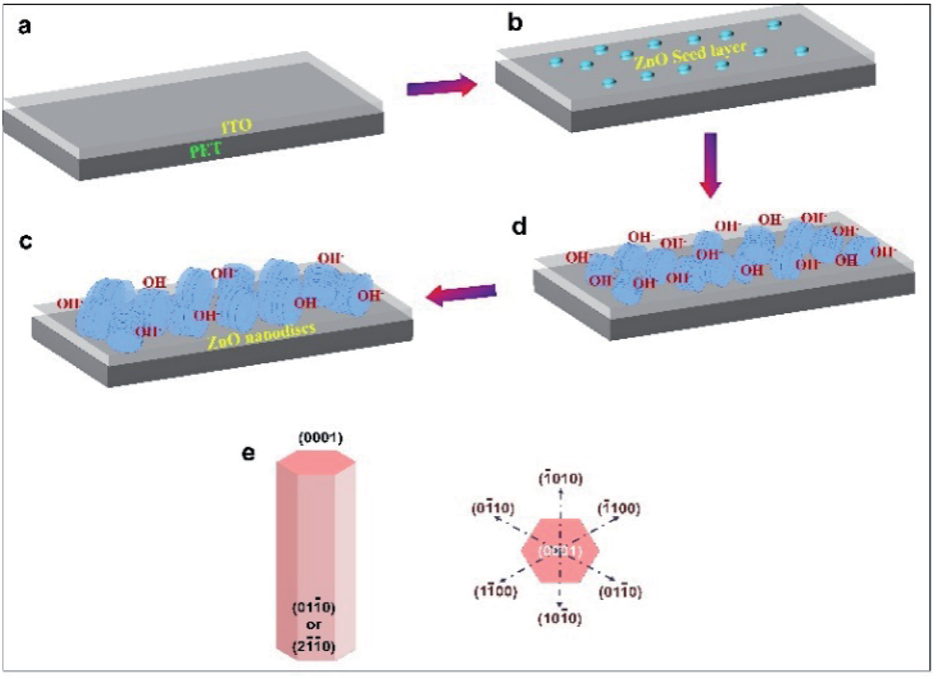
(a–e) Schematic of the formation of vertically aligned 2D piezoelectric ZnO nanodiscs on the flexible ITO/PET substrate.

The growth mechanism of ZnO nanodiscs can be understood by following the chemical reaction mechanism. Usually, the growth direction of ZnO crystals is along the [0001] direction due to the higher growth rate compared to other growth facets. In the seed assisted chemical solution route, crystal growth of ZnO nanostructures occurs in a two step process of nucleation and growth.^25^ The growth pattern of ZnO is influenced by several factors such as the Zn source, pH, nucleation conditions, presence of a complexing agent and the internal structure of the ZnO crystal.^26^ It is reported that formation of ZnO nanostructures is significantly affected by the presence of polar surfaces. The oppositely charged ions in ZnO produce positively charged Zn-(0001) and negatively charged O-(0001̄) surfaces, resulting in the formation of a normal dipole moment and spontaneous polarization along the *c*-axis and so preferential growth of ZnO along the *c*-axis is energetically favourable, resulting in the formation 1D nanostructures.^27^ However, in the present study, the formation of 2D like ZnO nanodiscs is due to a highly dense ZnO seed growth layer obtained by several cycles (∼10) of spin coating on the flexible ITO/PET substrate and its subsequent annealing at an adequate temperature of 150 °C. The schematic representation for growth of vertically aligned ZnO nanodiscs on the flexible ITO/PET substrate is shown in Fig. 3a–d. It is proposed that multiple seed layer coating cycles lead to formation of a thick ZnO seed layer. In the main chemical reaction process, OH^−^ ions are released from HMTA, which binds to the polar surface of seed crystallites. We proposed that the adherence/binding of extra OH^−^ ions onto the polar surface of ZnO suppressed the preferential growth of ZnO along the *c*-axis and allowed the lateral growth of ZnO. Moreover, the growth mechanism of nanodiscs may also be understood in terms of favourable growth direction, growth facets and the shielding effect due to OH^−^ ions. The surplus OH^−^ ions may preferentially adsorb on the positively charged (0001) Zn-terminated surface and the growth rates along the [01−10] and [2−1−10] directions are increased to a certain extent due to the shielding effect of OH^−^ ions on the (0001) surface of ZnO; thus the highest growth rate along the [0001] direction and the larger growth facets of (2−1−10) and (01−10) promote the formation of ZnO nanodiscs (Fig. 3e).^16,25^ To further understand the growth mechanism of ZnO nanodiscs, a control experiment for the growth of ZnO nanostructures was performed and FE-SEM and XRD analyses of the ZnO sample grown on a ZnO seed layer coated for five cycles on the flexible ITO/PET substrate were performed and the results are shown in Fig. S2a and b.† The FE-SEM and XRD results reveal the formation of a well defined one dimensional (1-D) ZnO nanorod-like morphology with preferred growth along the *c*-axis. These results also support the proposed growth mechanism of ZnO nanodiscs.

The Fourier-transform infrared spectroscopy (FT-IR) spectrum of ZnO nanodiscs grown on the ITO/PET substrate is presented in Fig. S3.† The FTIR spectrum was recorded in the range of 400–4000 cm^−1^. Fig. S3† shows that ZnO nanodiscs exhibited different absorption bands in various frequency regions. An absorption band for the stretching mode of Zn–O was observed below 1000 cm^−1^.^28^ The IR band appearing at 429 cm^−1^ corresponds to the bending vibration absorption of the Zn–O bond.^29^ The IR bands for the PET substrate were also observed in the spectrum. The absorption bands appearing at frequencies of 732 and 873 cm^−1^ are due to out of plane vibrations of the benzene group of PET, while the bands detected at 1017 and 1342 cm^−1^ are due to in-plane vibrations of benzene and CH_2_ wagging of glycol, respectively.

To fabricate the piezoelectric nanogenerator, a thin CNT–PDMS film was also fabricated. The morphology of the as-fabricated CNT–PDMS layer was also investigated from the FE-SEM image (Fig. S4a).† It is clearly seen that CNTs are randomly distributed throughout the PDMS matrix. Moreover, the fabricated CNT–PDMS polymer composites were also characterized through FT-IR and the obtained spectrum is shown in Fig. S4b.† The FT-IR spectrum depicts all the characteristic peaks of PDMS and CNTs. The peaks at 2961 and 2905 cm^−1^ appeared due to CH_3_ asymmetric and symmetric stretching, respectively. The peak appearing at 1257 cm^−1^ is due to the CH_3_ symmetric bond bending while the peak observed at 1010 cm^−1^ is assigned to Si–O–Si asymmetric bond stretching.^30^ The IR band at 1412 cm^−1^corresponds to C=C stretching while the peak at 787 cm^−1^ appeared due to C–H bending.^31^ Moreover, the electrical conductivity of the fabricated CNT–PDMS film layer was measured and found to be 9.4 mS m^−1^.

**Fig. 4:**
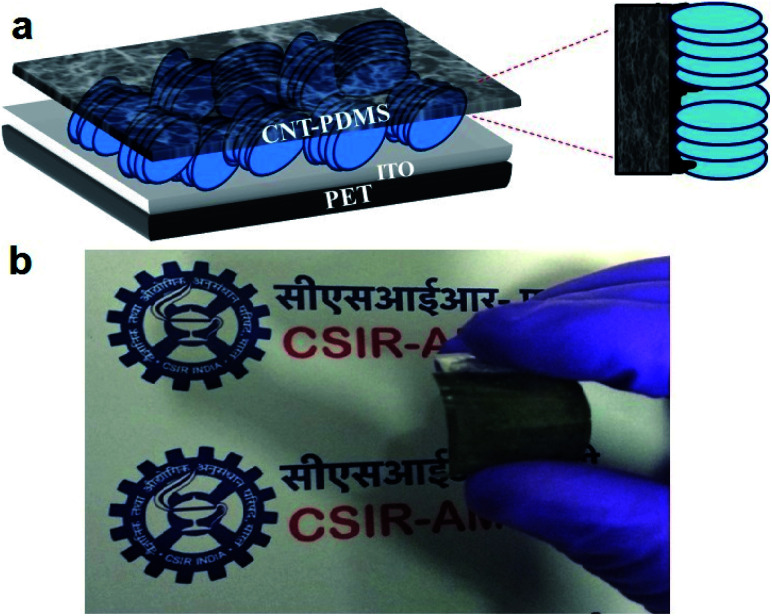
(a) Schematic representation of the fabricated ZnO nanodisc based device with ITO as the base electrode and the CNT–PDMS polymer composite as the top electrode and (b) original photograph of the flexible nanogenerator device with the CNT–PDMS layer.

The vertically aligned ZnO nanodisc based piezoelectric nanogenerator was fabricated using the CNT–PDMS as the top electrode and the schematic and original images of the flexible device are shown in Fig. 4.

**Fig. 5:**
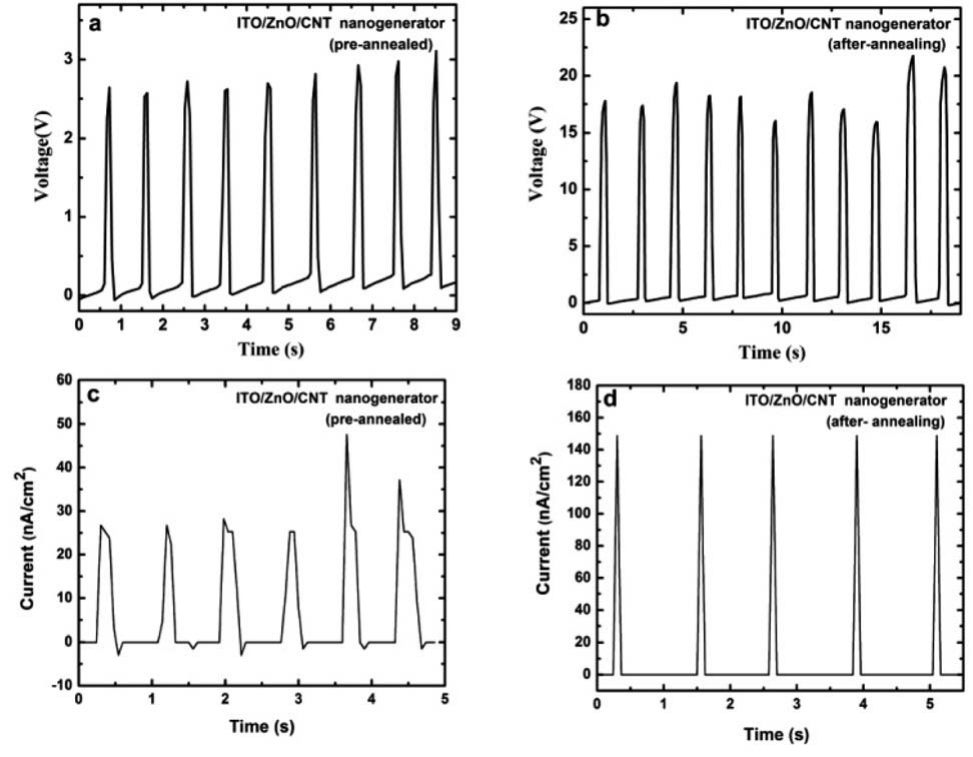
(a and b) Output voltages and (c and d) output current density experimentally obtained from the nanogenerator under vertical compressive strain before and after thermal annealing.

The piezoelectric output potential of the flexible vertically aligned ZnO nanodisc based nanogenerator was measured under vertical compressive stress (∼0.1 kgf). The piezoelectric output voltage measured for the device is shown in Fig. 5a. An output voltage of 2.5 V and output current of 30 nA cm^−2^ were detected under vertical mechanical stress. The output power generated from the ZnO nanodiscs was about 75 nW cm^−2^. A switching polarity test was also conducted and an electric signal with opposite polarity was observed when the device was connected in reverse bias mode (Fig. S5).† In order to confirm that the output voltage/current are generated due to the piezoelectric properties of the 2D ZnO nanodiscs, a separate device was fabricated without ZnO nanodiscs using ITO/PET and the CNT–PDMS layer. No significant output voltage was detected from the device (data shown in Fig. S6),† confirming that the output electric signals generated from the ZnO nanodisc based nanogenerator are due to the piezoelectric properties of ZnO.

It is worth pointing out that, although ZnO nanowires/nanorods exhibit better piezoelectric properties, the piezoelectric charge coefficient increases with the increase of the aspect ratio.^16^ It is noted that despite having 2D morphology, the output voltage from 2D ZnO nanodisc based nanogenerators was high as compared to that from previously reported 1D ZnO nanorod based nanogenerators.^15,32^ The high output voltage may be due to the use of the CNT electrode which enhances the contact of ZnO nanodiscs with the CNT electrode, since CNT layers have a nanosized network surface with nanopores which promote the contact with vertical nanodiscs. It is interesting to note that the fabricated device exhibited a DC type electric signal rather than an AC type electric signal which may be due to the presence of OH^−^ ions. In the absence of any external pressure, negative OH^−^ ions are present at the top surface of ZnO nanodiscs (towards the top electrode) and when an external vertical force is applied on the device, a negative piezoelectric potential is created on the top side and a positive piezoelectric potential is induced on the bottom side of the nanogenerator. Therefore, piezoelectric potential driven electrons are moved *via* the external circuit and collected at the interface between the top electrode and the top side of nanodiscs. As a result, a positive pulse was generated while applying pressure on the device. However, when the compressive force was removed, the piezoelectric potential disappeared and due to the presence of negatively charged (OH^−^) ions, the back flow of accumulated electrons was restricted due to the electrostatic repulsive force, and no piezoelectric signal appeared during the releasing force. As a result the DC type electric signal was generated from the vertically aligned ZnO nanodiscs.^16,25^

To further improve the output voltage from the flexible vertically aligned ZnO nanodisc based nanogenerator, the sample was thermally annealed at 150 °C for 2 hours. The output voltage and current of the thermally annealed device were measured under the same compressive strain and the output results are shown in Fig. 5b.

**Fig. 6:**
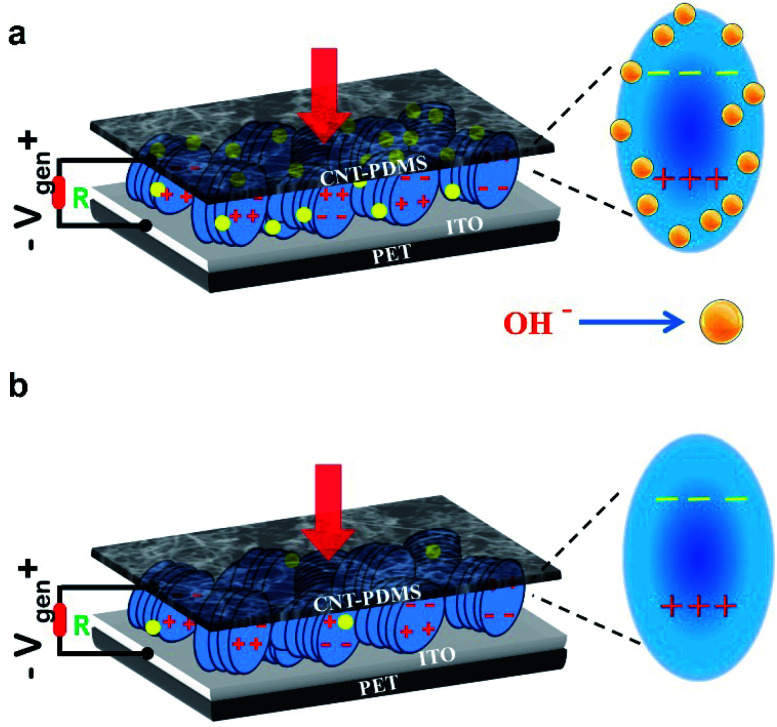
Schematic diagram of (a) the working mechanism of the piezoelectric nanogenerator under vertical pressure and (b) enhancement of the piezoelectric output voltage due to thermal annealing and due to reduction of OH^−^.

The output results show that the thermal annealing can significantly increase the output voltage as compared to that for the pristine ZnO nanodisc based nanogenerator. It is noted that the thermally annealed device showed an average output voltage of 17 V under the same stress, which is almost 8 times higher than that of the pre-annealed ZnO nanodisc based nanogenerator device. It is worth noting that the pre-annealed nanogenerator device exhibits an output current density of about 30 nA cm^−2^ and a very high current density of 150 nA cm^−2^ was achieved from the thermally annealed nanogenerator under the same compressive strain (Fig. 5c and d). The output power generated from the annealed ZnO nanodisc based nanogenerator was around 2.55 μW cm^−1^,^2^ which is approximately 34 times higher than that from the pre-annealed ZnO nanodisc based nanogenerator (75 nW cm^−2^). The dramatic increase in the output voltage and current can be understood from the schematic image of Fig. 6. It is noted that the piezoelectric potential is reduced due to the screening effect caused by free electrons and surface defects in the pristine ZnO nanostructures. Since pristine ZnO nanostructured surfaces are usually very rich in defects, predominantly oxygen vacancies, which act as electron donors, surface passivation due to thermal annealing can eliminate/suppress these surface/oxygen defects, which results in a high piezoelectric potential (as shown in Fig. 6).^33–35^ A stability test of the device was carried out and an almost stable output voltage with an average value of ∼17 V was obtained (Fig. S7).† The output performance of the developed ZnO nanodisc based nanogenerator was compared with that of previously reported ZnO based nanogenerators. The output performance was much higher than that of other ZnO nanostructure/film based nanogenerators (Table 1).

**Table 1:** Output performance comparison with previous reports

Sr. no.	Material	Electrode	Output voltage (V)	Output current/current density	References
1	ZnO nanorods	Au/Cr	0.25 V	100 nA cm^−2^	36
2	Au@ZnO nanorods	Au/Cr	2 V	1 μA cm^−2^	36
3	Zno nanorods	Ag	10 mV	10 nA	37
4	NiO–ZnO heterostructure	Ni, Al	430 mV	40 nA cm^−^^2^	38
5	InN nanowire	Si, Au	0.055 V	211 nA	39
6	ZnO nanosheet	Al, Cu	0.924 V	6 μA	40
7	ZnO nanodisc	CNT : PDMS	17 V	150 nA cm^−2^	Present work

It is interesting to observe the direct current (DC) type output voltage from the vertically aligned ZnO nanodisc based nanogenerator under a vertical compressive force, which added an advantage by eliminating the requirement for a rectification circuit to convert the alternating current (AC) signal to a DC signal. The piezoelectric output performance of the piezoelectric nanogenerator also depends on the dielectric constant of the piezoelectric material. The dielectric constant and dielectric loss of ZnO nanodiscs were investigated in the frequency range of 20 Hz to 2 MHz at room temperature using a high precision LCR meter and the value of the dielectric constant was evaluated using the equation:
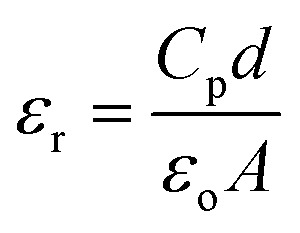
where *C*_p_ is the capacitance, *ε*_o_ is the permittivity of free space, and *d* and *A* are the thickness and area of the sample, respectively.^41^

**Fig. 7:**
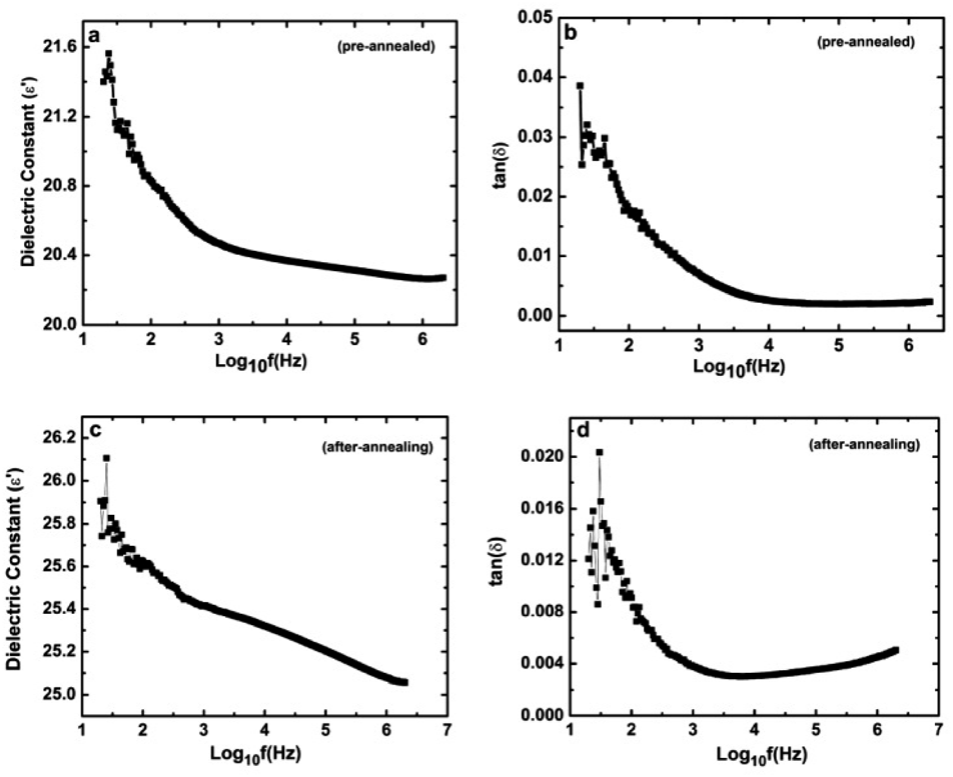
Variation of (a) dielectric constant and (b) dissipation factor of pre-annealed ZnO nanodiscs and (c) variation of the dielectric constant and (d) dissipation factor of thermally annealed ZnO nanodiscs with frequency.

The surface of the sample was coated on both sides with conducting silver paste to form the parallel plate capacitor. Fig. 7a shows the variation of the dielectric constant with applied frequency. It is observed that the dielectric constant decreases with increasing frequency and a dielectric constant of 21.5 was observed at 20 Hz and it reached a value of 20.3 at 2 MHz. Such dielectric behavior of the ZnO nanodiscs may be attributed to contribution from different kinds of polarization such as electronic, ionic, dipolar, orientation and space-charge polarization.^42–45^ The dielectric loss tangent (tan *δ*) of the ZnO nanodisc (Fig. 7b) also shows a similar trend to that of the dielectric constant and reached a low value of 0.005 at 2 MHz. The observed dielectric properties extend the scope of 2D ZnO nanodiscs for large scale applications in optoelectronics devices and high performance electronic devices.

Variation in the dielectric constant and dielectric tangent loss of the thermally annealed sample was also measured in the frequency range of 20 Hz to 2 MHz and is shown in Fig. 7c and d. It is interesting to observe that the value of the dielectric constant increased and reached 26 at 20 Hz. The increase in the dielectric constant may be attributed to the decrease in conductivity due to a reduction in oxygen vacancies and OH^−^ ions, which results in a high piezoelectric output from the thermally annealed ZnO nanodisc based nanogenerator. The above results confirm that the 2D material and 2D ZnO nanodisc based nanogenerator has great advantages compared traditional materials for developing flexible energy devices for powering next generation large array 2D devices, advancing its applications as future wearable and flexible low-cost electronic devices.

## Experimental

### Synthesis and fabrication of 2D-ZnO nanodisc based piezoelectric nanogenerators

Synthesis of ZnO nanodiscs was carried out through a low temperature cost effective aqueous solution method. A seed solution was prepared by mixing 0.04 M zinc acetate dihydrate [Zn(CH_3_COO)_2_·2H_2_O] into ethanol. The seed solution was spin coated on the flexible ITO/PET substrate at 1500 rpm for 60 seconds. The resultant seed layer was dried on a hot plate at 150 °C for 10 minutes and the process was repeated 10 times. To grow the vertically aligned ZnO nanodiscs, an aqueous solution of zinc nitrate hexahydrate [Zn(NO_3_)_2_·6H_2_O, 0.025 M], HMT [hexamethylenetetramine (CH_2_)_6_N_4_, 0.025 M] and deionized water was prepared and the seed layer coated on the ITO/PET substrate samples was immersed into the solution at 90 °C temperature for 3 hours. The top electrode of the ZnO nanodevice was fabricated using carbon nanotubes and the PDMS polymer. CNTs and PDMS were homogeneously dispersed together and a flexible composite layer was obtained by spin coating the obtained mixture on the ITO/PET substrate at 1000 rpm for 30 seconds. Fabrication of the 2D-ZnO nanodisc based piezoelectric nanogenerators was carried out by mechanical integration of the fabricated CNT : PDMS based conductive layer onto vertically aligned ZnO nanodiscs.

### Characterization and measurements

The crystalline phase and structure were measured using the X-ray diffraction (XRD) technique (Rigaku Mini Flex II) with Cu K_α_ radiation (*λ* = 1.5401 Å). The morphology of the grown nanostructure was identified using a field emission scanning electron microscope (FE-SEM). High resolution-transmission electron microscope (HR-TEM) micrographs of the ZnO nanodisc were obtained using a high resolution JEOL JEM-2100F microscope. Elemental study was performed through energy dispersive spectroscopy using FE-SEM. Fourier-transform infrared spectroscopy (FT-IR) spectra of the ZnO nanodiscs were recorded in the range 400–4000 cm^−1^ at a 4 cm^−1^ resolution using a Thermo Scientific Nicolet iS50 FT-IR spectrometer. The dielectric constant and tangent loss of the nanodiscs were measured using a Keysight LCR meter (E4980A) in the frequency range 20 Hz to 2 MHz. The piezoelectric output performance of the ZnO nanodisc based nanogenerator device was determined through a Keithley (6517B) system electrometer (impedance > 200 TΩ).

## Conclusion

In conclusion, we developed a flexible vertically aligned 2D ZnO nanodisc based piezoelectric nanogenerator using a conducting CNT–PDMS composite film as the top electrode. X-ray diffraction spectroscopy and electron microscopy studies confirmed the formation of vertically aligned ZnO nanodiscs on the ITO/PET substrate. The output voltage and current density of the pristine ZnO nanodisc based nanogenerator were measured and found to be 2.5 V and 30 nA cm^−2^, respectively. The output performance of the pristine 2D ZnO nanodiscs was dramatically improved by thermal annealing of vertically aligned ZnO nanodiscs. A significant increase of 8 fold in the output voltage and a 5 fold enhancement in the output current were achieved. An enhancement of the dielectric properties and piezoelectric output performance of synthesized nanodiscs were discussed in terms of suppression of surface/oxygen defects in the pristine ZnO nanostructures. The present results indicate an effective way to design the 2D based DC type nanogenerator and to improve the performance of piezoelectric nanogenerators without any doping for their potential application in next generation 2D piezoelectric areas.

## Conflicts of interest

There are no conflicts to declare.

## Supplementary Material

NA-002-C9NA00789J-s001
