# Supplementary material for: A high performance flexible two dimensional vertically aligned ZnO nanodisc based piezoelectric nanogenerator via surface passivation
Source: Nanoscale Adv. 2020 Mar 20;2(5):2044–51. doi: 10.1039/c9na00789j (PMC9418489; doi:10.1039/c9na00789j)
Supplement: NA-002-C9NA00789J-s001 [file NA-002-C9NA00789J-s001.pdf]

Electronic Supplementary Material (ESI) for Nanoscale Advances.
This journal is © The Royal Society of Chemistry 2020

Supporting Information

High performance flexible two dimensional vertical aligned ZnO nanodiscs based piezoelectric nanogenerator via surface passivation.

Ketki Verma,[a] Dhiraj Kumar Bharti,[a, b] Simadri Badatya, Avanish Kumar Srivastava,[a,b,] Manoj Kumar Gupta[a,b,*]

[a]*Advanced Construction Materials Division, CSIR-Advanced Materials and Processes Research Institute,* [b]*Academy of Scientific and Innovative Research (AcSIR), Bhopal, Madhya Pradesh, India-462026.*

*E-mail: mkgupta@ampri.res.in , manojampri@gmail.com .

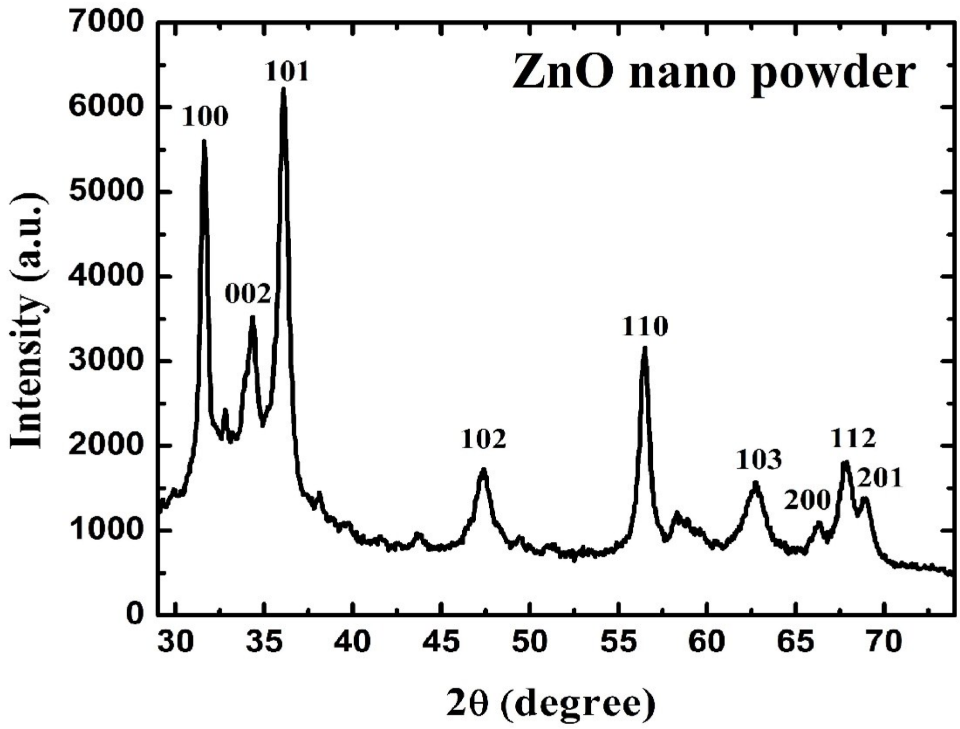

Figure S1. XRD spectrum of ZnO nanodiscs powder collected from ITO/PET substrate

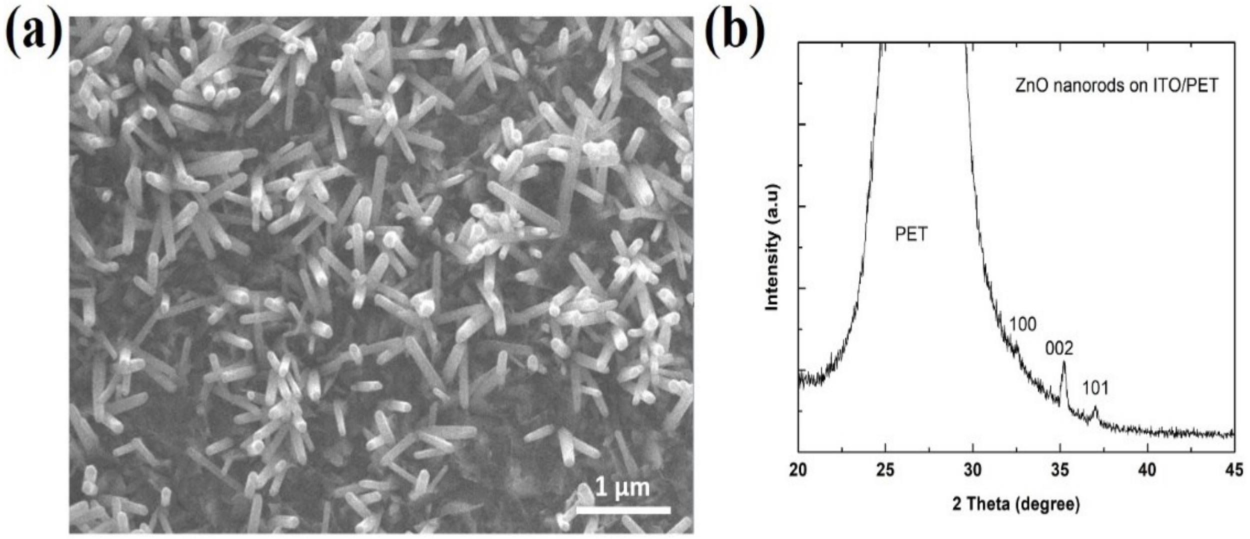

FIG S2. (a) FE-SEM and (b) XRD of pure ZnO nanorods grown in ITO/PET substrate (5 times seed layer coating)

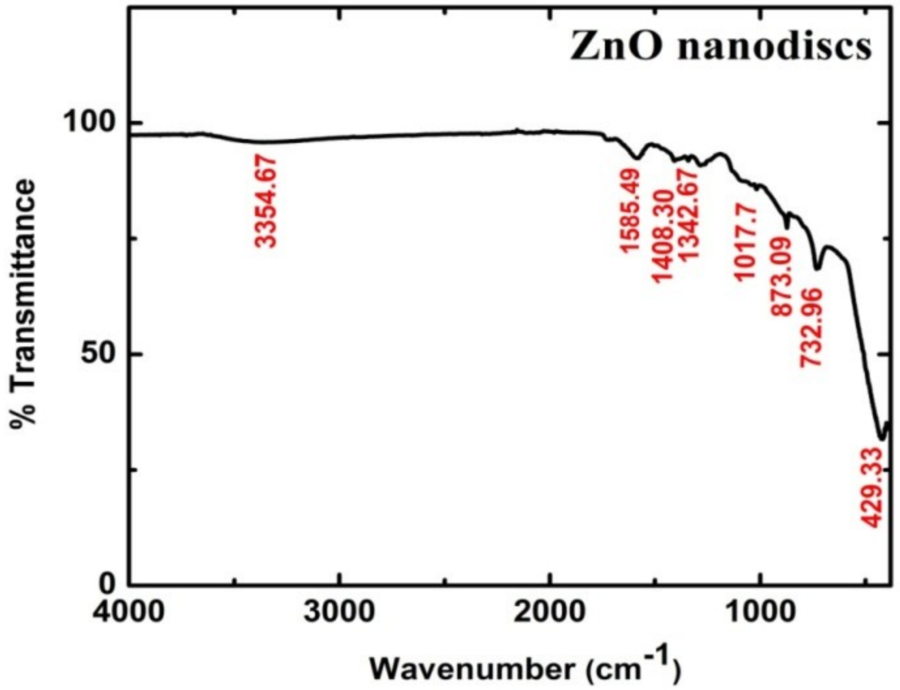

FIG. S3 FT-IR spectra of ZnO nanodiscs on ITO/PET substrate recorded in the range of 400-4000 cm^{-1}

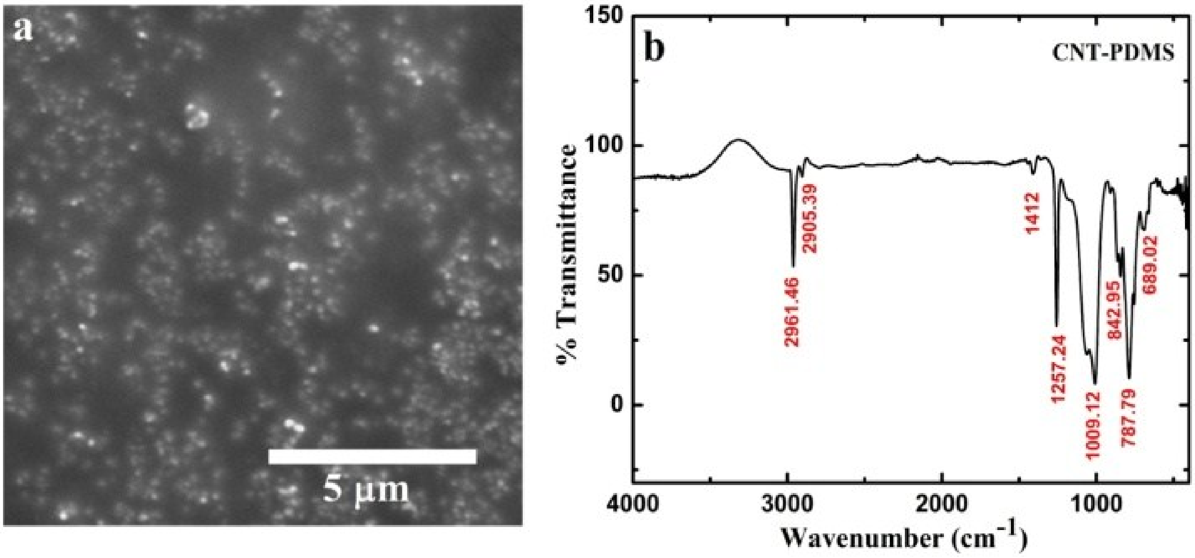

FIG. S4 (a) FE-SEM image of CNT: PDMS composite layer, (b) FT-IR spectra of CNT: PDMS composite layer in the range of 400-4000 cm1.

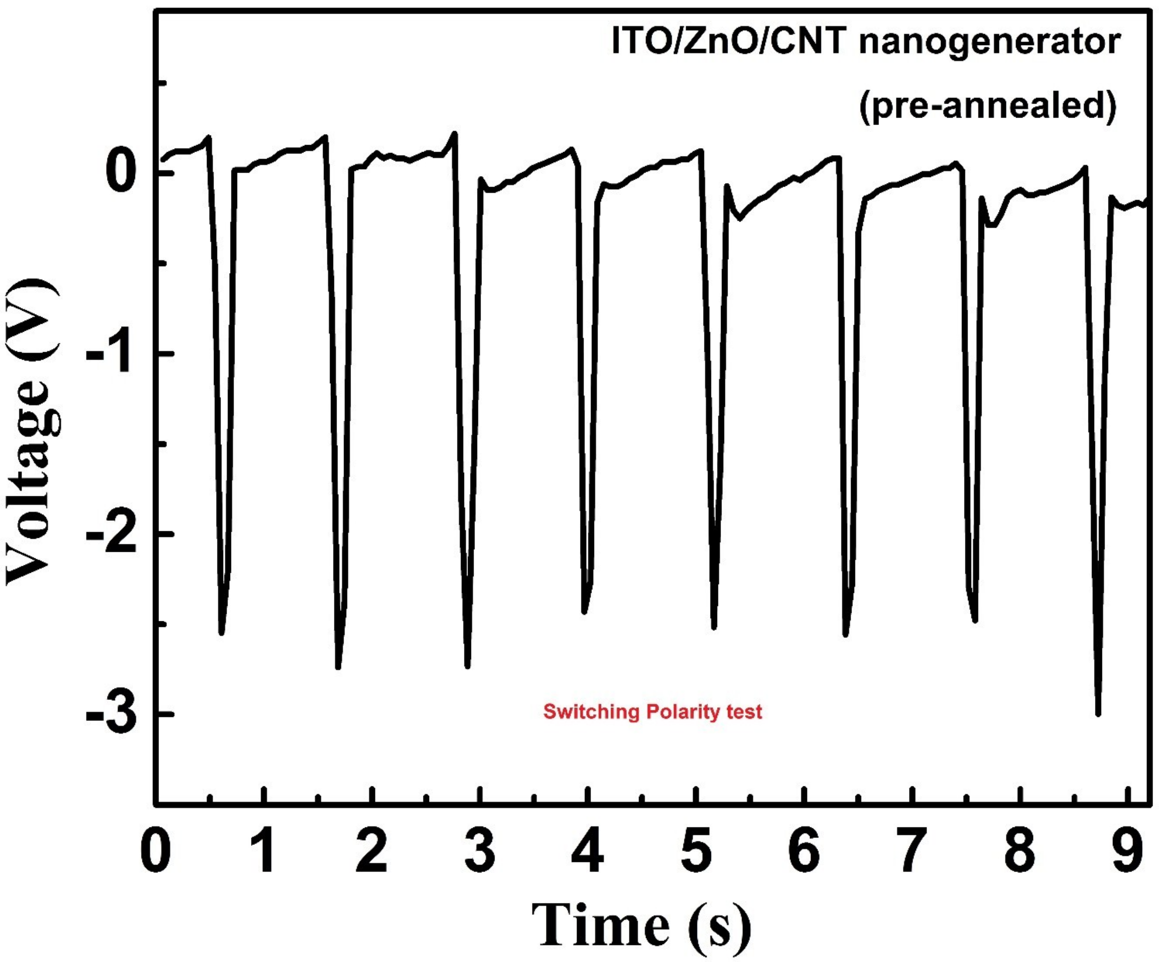

FIG. S5 Switching polarity test of 2D-ZnO nanodiscs based nanogenerator

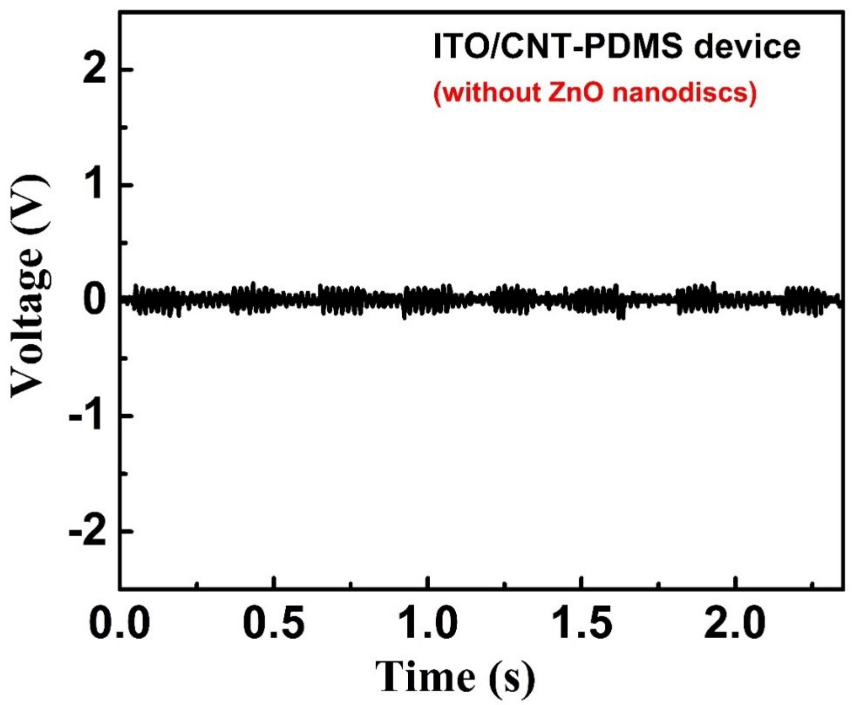

FIG. S6. Piezoelectric output voltage from ITO and CNT-PDMS device (without 2D-ZnO nanodiscs) based nanogenerator, showing no significant electric output

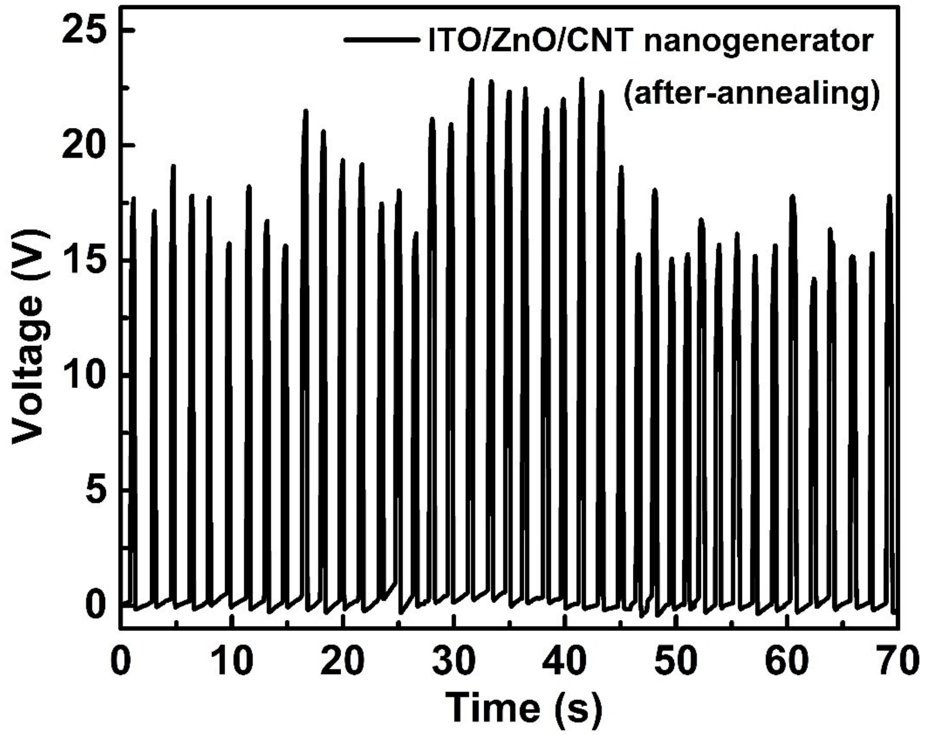

FIG. S7. Stability data (output voltage) of the ZnO nanodiscs based nanogenerator.
